# Serial evaluation of the SOFA score is reliable for predicting mortality in acute severe pancreatitis

**DOI:** 10.1097/MD.0000000000009654

**Published:** 2018-02-16

**Authors:** Yu-San Tee, Hsin-Yueh Fang, I.-Ming Kuo, Yann-Sheng Lin, Song-Fong Huang, Ming-Chin Yu

**Affiliations:** a Division of General Surgery, Department of Surgery, Chang Gung Memorial Hospital, Kweishan, Taoyuan city, Taiwan; b Department of Surgery, Xiamen Chang Gung Hospital, Xiamen, Fujian, China.

**Keywords:** mortality, pancreatitis, scoring system, Sequential Organ Failure Assessment, sequential scoring

## Abstract

Acute severe pancreatitis caused high mortality, and several scoring systems for predicting mortality are available. We evaluated the effectiveness of serial measurement of several scoring systems in patients with acute severe pancreatitis.

We retrospectively obtained serial measurements of Ranson, Acute Physiology and Chronic Health Assessment (APACHE) II, and Sequential Organ Failure Assessment (SOFA) scores of 159 patients with acute severe pancreatitis.

The overall mortality rate was 20%, and early mortality (in the first 2 weeks) occurred in 10 (7.4%) patients, while late mortality occurred in 17 (12.6%).

All scoring systems were reliable for predicting overall and intensive care unit mortality, while the SOFA score on day 7 presented the largest area under the receiver operator characteristic (ROC) curve (0.858, SE 0.055). Changes in scores over time were evaluated for predicting the progression of organ failure, and the change in SOFA score on hospital day 7 or no interval change in SOFA score was associated with higher mortality rates.

APACHE II and SOFA scores are both sensitive for predicting mortality in acute pancreatitis. The serial SOFA scores showed reliable for predicting mortality. Hospital day 7 is a reasonable time for SOFA score reassessment to predict late mortality in acute severe pancreatitis.

## 1 Introduction

Acute pancreatitis is the leading gastrointestinal cause of hospitalization in the United States,^[1]^ and the number of cases is growing in European countries due to increasing alcohol consumption.^[2]^ In the past 2 decades, the pathogenesis of acute pancreatitis has been well-studied, treatment guidelines have been developed, and patient outcomes have improved.^[3–8]^ However, the mortality rate in patients with acute pancreatitis remains approximately 10% and is reportedly as high as 30% with severe disease.^[2,9–13]^ Two peaks in mortality have been reported in patients presenting with severe acute pancreatitis. Early death usually occurs as a result of multiple organ dysfunction syndrome (MODS) due to systemic inflammatory response syndrome (SIRS) caused by the release of various cytokines in the first 2 weeks.^[9,10,12,14–18]^ Approximately half of patients die 2 weeks later due to peripancreatic necrosis, infection, and secondary MODS.^[12,14]^

Various outcome scoring systems have been proposed to predict the prognosis of patients with acute pancreatitis. These include the Ranson,^[19]^ Acute Physiology and Chronic Health Assessment (APACHE) II and III,^[5,20,21]^ and Sequential Organ Failure Assessment (SOFA) scoring systems.^[22–27]^ However, scoring only a single time ignores many factors that can influence the outcome during the course of the illness. The measurements from these scoring systems have been shown to more effectively represent the dynamic changes of critically ill patients.^[28–31]^ In addition, the SOFA scoring system has been shown to perform better and to be easier to apply.^[25]^ However, to our knowledge, there has not been a study evaluating serial measurements from the scoring systems in patients presenting with acute pancreatitis.

The aim of this study was to evaluate the efficacy and reliability of serial SOFA scores for predicting mortality of patients presenting with acute pancreatitis.

## 2 Methods

This was a single-center retrospective study performed at Chang Gung Memorial Hospital, Linkou Branch in North Taiwan. This study included a cohort of patients diagnosed with acute pancreatitis and admitted to the intensive care unit (ICU) between January 2005 and December 2010. The diagnosis of acute pancreatitis was based on clinical presentation, laboratory parameters, and radiographic evidence (ultrasonography or computed tomographic scan imaging). Of the 159 patients evaluated for participation in the study, 24 were excluded for having a history of chronic pancreatitis, an ICU stay less than 48 hours, traumatic pancreatitis, pancreatitis resulting from surgical complications, or being referred from another institution. The remaining 135 patients were included in the study. They were divided into 2 groups: survivor (n = 108, 80.0%) and nonsurvivor (n = 27, 20.0%), and the nonsurvival group was further divided into 2 subgroups: early (≤14 days, n = 10) and late (>14 days, n = 17) mortality.

Demographic data, including patient characteristics, etiology of pancreatitis, organ support systems used, length of ICU stay, and duration of total hospital stay, were recorded. The severity of illness was measured via Ranson, APACHE II, and SOFA scores. To evaluate the efficacy of serial scores, the APACHE II score was recorded on admission and after 48 hours. The SOFA score was recorded on admission; after 48 hours; and on days 7, 14, and 21.

Statistical analysis was performed using IBM SPSS statistics 22.0, IBM Cororation, North Castle drive, Armonk, USA. Continuous variables were analyzed with *t* test or 1-way analysis of variance (ANOVA), while categorical variables were compared using a Chi-square test. A *P* value < .05 was considered significant for the analysis. The efficacy of scoring systems for predicting mortality rates in pancreatitis was explored using receiver operator characteristic (ROC) curves and the area under ROC curves (AUROC).

## 3 Ethics

This study was approved by the institution's ethics committee (IRB no.101–2942B), which waived the requirement to obtain informed consent.

## 4 Results

Patient characteristics are summarized in Table 1. The study cohort consisted primarily of men (72.6%). The most common etiology of acute pancreatitis was alcoholism (43.7%) followed by biliary pancreatitis (34.8%). The overall mortality rate was 20%, and most patients died while in the ICU (17%). A total of 10 (7.4%) patients died within 14 days, while 17 (12.6%) died 14 days later. The most commonly used organ support system was mechanical ventilation (48.1%) followed by vasoactive agents (27.4%), renal replacement therapy (14.1%), and extracorporeal membrane oxygenation (ECMO) (2.2%). There were 110 patients in the survival group and 28 in the nonsurvival group. The nonsurvival group was significantly older (*P* < .001) and required organ support systems more often (*P* < .05) than the survival group. Patients in the nonsurvival group also had a longer ICU stay (29.2 ± 38.3 vs 8.1 ± 13.1 days, *P* = .000) and hospital stay (50 ± 73.6 vs 29.1 ± 26.9 days, *P* = .018) than those who survived. Serial measurements from all scoring systems in the nonsurvival group were significantly higher than in those who survived (Table 2).

**Table 1:**
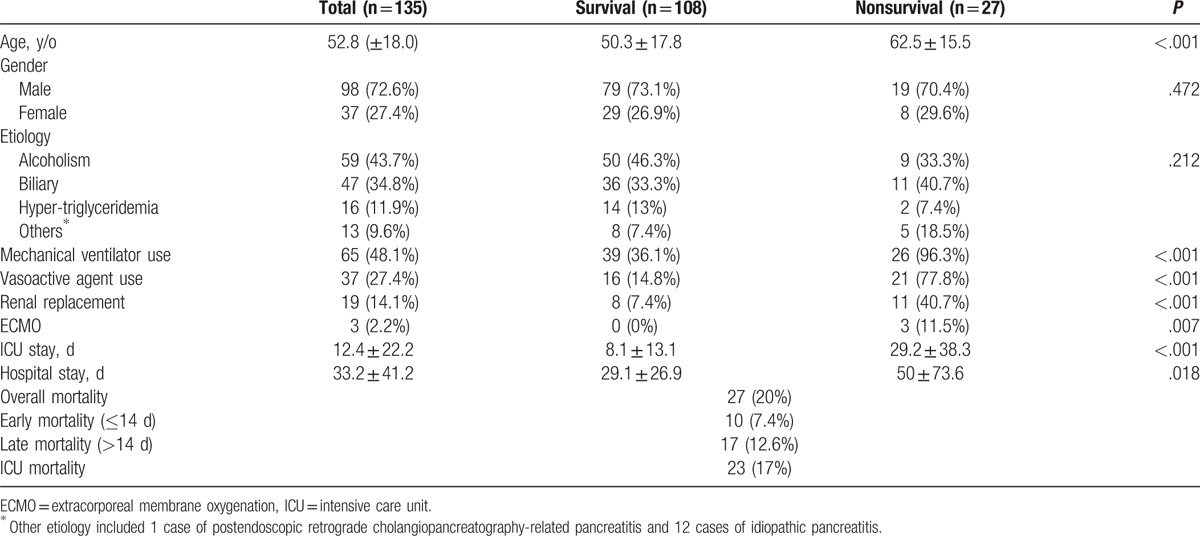
Demographics of study population.

**Table 2:**
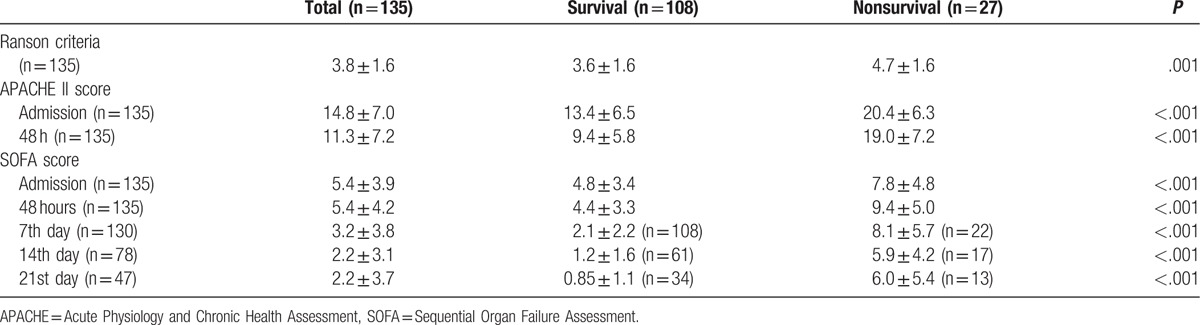
Comparison of scoring systems in survivors and nonsurvivors.

**Figure 1:**
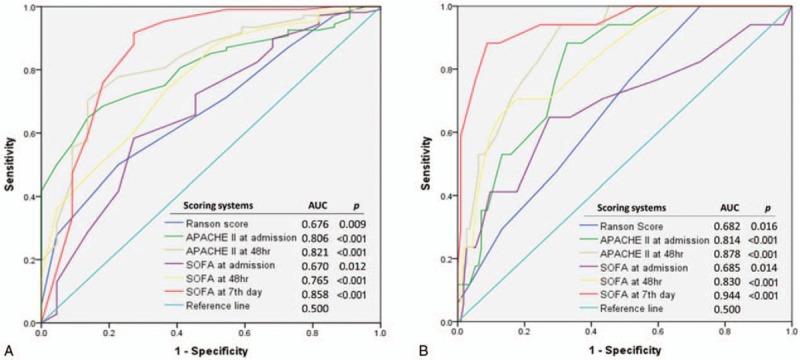
Comparison of the area under the receiver operating characteristic curve for predicting (A) overall mortality and (B) ICU mortality in patients presenting with acute pancreatitis. APACHE = Acute Physiology and Chronic Health Assessment, ICU = intensive care unit, SOFA = Sequential Organ Failure Assessment.

The area under the ROC (AUROC) curves for prediction of overall mortality and ICU mortality in patients presenting with acute pancreatitis are shown in Fig. 1A and 1B, respectively. All scoring systems in this study were reliable for predicting overall mortality and ICU mortality. The SOFA score on hospital day 7 had the largest AUROC (0.858, SE 0.055 and 0.944, SE 0.030, respectively, *P* < .001). The AUROC for the APACHE II score on admission and at 48 hours were both greater than 0.80 for the prediction of both overall and ICU mortality. Furthermore, 3 scoring systems were compared at admission for overall mortality and all were significant to predict patients’ outcome, but APACHE II had the largest AUROC (0.806, SE 0.041, *P* < .001). In serial evaluation, APACHE II at 48 hours and SOFA score at day 7 both were good at prediction. (0.821 and 0.858, respectively, *P* < .001).

**Figure 2:**
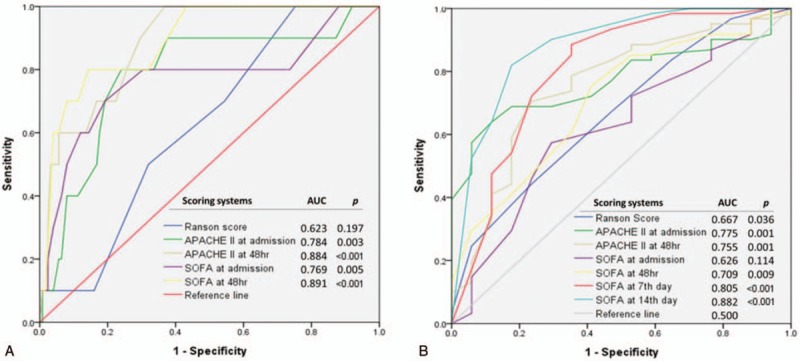
Comparison of the area under the receiver operating characteristic curve for predicting (A) early mortality and (B) late mortality in patients presenting with acute pancreatitis.

**Figure 3:**
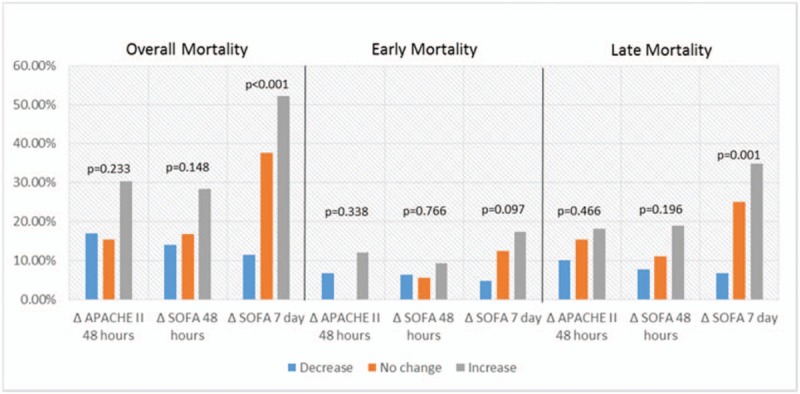
Changes in APACHE II and SOFA scores in relation to mortality. APACHE = Acute Physiology and Chronic Health Assessment, SOFA = Sequential Organ Failure Assessment.

The nonsurvival group was further analyzed after being divided into 2 subgroups, early (≤14 days) and late (>14 days) mortality. Figure 2 shows the comparison of the AUROC curves for prediction of early (a) and late mortality (b) in this study. The APACHE II score 48 hours after admission (0.884, SE 0.042) and the SOFA score 48 hours after admission (0.891, SE 0.047) had the largest AUROC for predicting early mortality. For predicting late mortality, the SOFA score on day 7 and day 14 both had an AUROC greater than 0.80 (0.805 and 0.882, respectively), while the other models were less than 0.80. Changes in scores over time from the scoring systems were evaluated (Fig. 3). The change in APACHE II and SOFA scores between admission and 48 hours after admission and the change in SOFA score between admission and day 7 of hospitalization were compared. Patients whose SOFA scores were unchanged or increased between admission and hospital day 7 had a significantly higher overall mortality rate (37.50% and 52.20%, respectively, *P* < .001) and higher late mortality rate (25% and 34.8%, respectively, *P* = .001).

## 5 Discussion

In the past 2 decades, the outcomes of patients with acute pancreatitis have improved, as guidelines have evolved and advances have been made in diagnostic and therapeutic interventions, especially in those with severe disease.^[3–8]^ However, severe acute pancreatitis is still associated with a 30% mortality rate. Cause of death in patients with acute pancreatitis varies based on timing: early death usually occurs as a result of SIRS leading to MODS, and late mortality occurs due to sepsis and its complications.^[14]^

Similar to western industrialized countries, alcohol and gallstones accounted for 78.5% of the cases of acute pancreatitis in our study.^[32,33]^ However, etiology had no influence on outcome. This may indicate that the pathogenic mechanism does not affect the course and outcome of acute pancreatitis. Some authors have published similar results.^[12,34]^ In addition to having higher scores on the scoring systems, we found that comorbid disease appears to be a poor prognostic factor in elderly patients with acute pancreatitis, which has also been seen in previous studies.^[11]^

The present study included patients with severe acute pancreatitis. The overall mortality rate in the study was 20%, with most deaths occurring in the ICU (17%). The mortality rate was lower than in previous studies because all patients are admitted to and treated in the ICU at Chang Gung Memorial Hospital, one of the tertiary medical centers in North Taiwan. Among these patients, 10 (37%) died within 2 weeks and 17 (63%) died after 14 days or more. This may indicate that advances in medical management have improved, so the treatment of early complications is more effective, which has reduced early mortality rate. However, late mortality still occurred in more than half of the patients. For this reason, more effort should be made in improving the methods for predicting late mortality in patients with acute pancreatitis.

The Ranson score, APACHE II score, and SOFA score are the most common scoring systems used in the ICU. These scoring systems were initially designed to predict mortality of critically ill patients during the first 48 hours. Modifications of the scoring systems and serial measurements have been proposed in the past decade and have been shown to be reliable for predicting mortality.^[28–31]^ In addition to obtaining Ranson and APACHE II scores on admission and at 48 hours, we obtained serial weekly measurements of SOFA scores (on admission; at 48 hours; and on days 7, 14, and 21) in this study. Nonsurvivals had significantly higher scores than survivors in all scoring systems. This was observed not only on admission, but it also continued until day 14 of the illness, indicating persistent organ failure, which carries a higher risk of mortality. We evaluated all prognostic predictors in this study and found that the most discriminatory AUROC in first 48 hours for predicting overall mortality were the APACHE II admission (AUC = 0.806) and APACHE II 48 hour (AUC = 0.821) scores. These 2 predictors also have the largest AUROC for predicting ICU mortality (AUC = 0.814 and 0.878, respectively). In this analysis, the Ranson score had the lowest AUROC for predicting mortality in patients with acute pancreatitis. These results parallel a previous meta-analysis by De Bernardinis et al,^[35]^ who found a poor predictive power associated with Ranson criteria. As the most widely studied scoring system for acute pancreatitis, the APACHE II score has been shown to have a good negative predictive value and modest positive predictive value.^[3,20]^ The present study confirms these results, but the complexity of the scoring system remains a challenge in daily clinical assessment.

In contrast to the APACHE II, SOFA provides an easier system for evaluating organ dysfunction using 6 reproducible variables that measure disease severity during an ICU stay.^[36]^ In our analysis, the SOFA score on admission and 48 hours after admission had smaller AUROC compared with the APACHE II score. But, interestingly, the SOFA score on day 7 was excellent for predicting overall mortality (AUC = 0.858) and ICU mortality (AUC = 0.944). Although the SOFA score obtained on day 7 has no role in early treatment plans, we evaluated it as a prognostic predictor of late mortality in acute pancreatitis and compared it with other models. It has a slightly smaller AUROC than the SOFA score on day 14, but reassessment on day 7 is more practical in clinical settings.

Organ failure is not static; it is a continuous process of alterations in organ function. It should not be evaluated at a single point in time. Some authors have suggested that changes in scores during treatment could be used to reflect treatment response and facilitate clinical decisions.^[28,37,38]^ In this analysis, we compared changes in APACHE II and SOFA scores over time. However, we looked at changes in these scores that occurred within 48 hours, which might be too short a period for guiding clinical decisions. Surprisingly, we found trends in the SOFA score over a period of 7 days were a sensitive indicator for late mortality.

**Figure 4:**
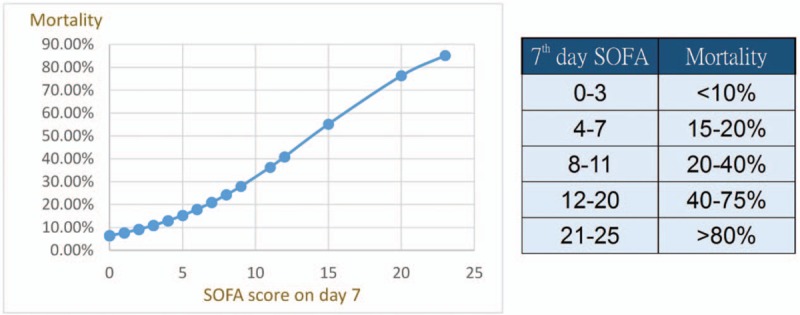
Correlation between SOFA score on day 7 and late mortality. SOFA = Sequential Organ Failure Assessment.

Serial evaluation of organ dysfunction has been evaluated and shown to be reliable in ICU practice. Using a variety of outcome predicting scoring systems, dynamic evaluation of the SOFA score has been shown to be a good prognostic indicator for critically ill patients.^[28,29]^ We first evaluated the reliability of serial evaluation using scoring systems in patients presenting with severe acute pancreatitis, and our results are similar to previously published reports. We also found the SOFA score on day 7 to be a reliable predictive model for late mortality in patients with acute pancreatitis (Fig. 4). By knowing the variation of scoring system scores over time, it may be possible to alter therapeutic decisions, which may result in a reduction in mortality rate.

This study is limited by its retrospective design. Also, using single-center data has well-known limitations. We only included patients with severe pancreatitis; however, populations may vary in disease severity between hospitals. Nonetheless, our center is an experienced and high-volume unit, so our data may be useful in other centers.

**Figure 5:**
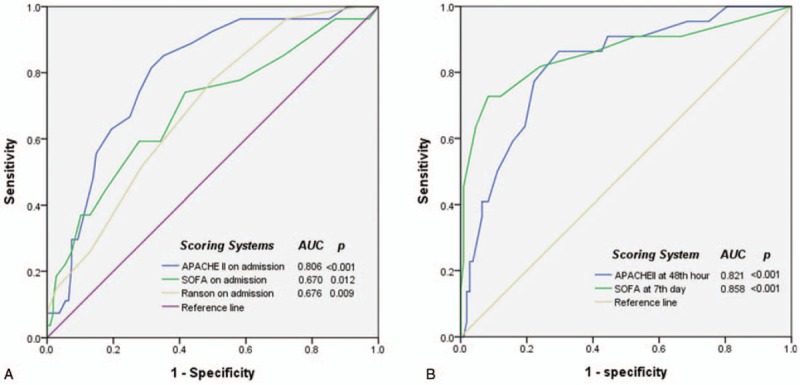
Comparison of AUROCs of scoring systems (A) on admission (B) interval follow-up for prediction of overall mortality in patients with acute pancreatitis. APACHE II score at 48th hour is one of the best prediction model currently, but APACHE II at 48 hours and SOFA score at day 7 both were good at prediction in serial evaluation.

In conclusion, serial SOFA scores were shown to be reliable for guiding clinical decisions and 1 week is a reasonable time for SOFA score reassessment to predict late mortality in acute pancreatitis. Although the APACHE II score and SOFA score are both sensitive for predicting mortality in acute severe pancreatitis, the SOFA score is an easier tool to apply in the ICU (Fig. 5).

## Acknowledgment

We thank Ms Yi-Ping Liu for administration management.
